# Nurse-Training, Small Hospitals, and the College of Nursing

**Published:** 1916-05-13

**Authors:** 


### THE EDITOR'S LETTER-BOX.

### Nurse-Training, Small Hospitals, and the College of Nursing.

*To the Editor of* The Hospital.

Sir,—I observe in that useful section of The Hospital, *The Institutional Worker*, for the 6th instant, under "Miscellaneous," that "Matron" raises the important question of the effect the College of Nursing will have on the training heretofore given to certain nurses at the smaller hospitals. This is an important question which might be usefully ventilated and considered on its merits from every point of view. Would you be willing to open your columns for a correspondence on these questions?—I am, Sir, yours faithfully. A Hospital Governor.

[We shall be very glad to open our columns to correspondence on this question, which is occupying much attention in the minds of many people connected with nursing in the United Kingdom at the present time. There is no doubt that the scheme of the College of Nursing provides means whereby full justice can, and we have no doubt will ultimately be done to the smaller schools. All who are connected with these schools and have practical knowledge could expedite matters by communicating their views to The Hospital, setting forth their present difficulties, their needs, the character and amount of work done, and some indication of the number of nurses who have been certificated by the smaller schools or some of them for a term of years. Dr. Hurd, the American authority, mentions this subject in his interesting article on page 153 of the present issue.—Ed. The Hospital.]

